# Puerarin Suppresses Invasion and Vascularization of Endometriosis Tissue Stimulated by 17β-Estradiol

**DOI:** 10.1371/journal.pone.0025011

**Published:** 2011-09-15

**Authors:** Dan Wang, Yuhuan Liu, Jie Han, Dongxia Zai, Mei Ji, Wen Cheng, Ling Xu, Luxi Yang, Miaoxia He, Jian Ni, Zailong Cai, Chaoqin Yu

**Affiliations:** 1 Department of Chinese Traditional Medicine, Changhai Hospital, Second Military Medical University, Shanghai, China; 2 Clinical Research Center, Changhai Hospital, Second Military Medical University, Shanghai, China; 3 Department of Obstetrics and Gynecology, Changhai Hospital, Second Military Medical University, Shanghai, China; 4 Department of Pathology, Changhai Hospital, Second Military Medical University, Shanghai, China; 5 Institute of Micro/Nano Science and Technology, Shanghai Jiaotong University, Shanghai, China; Clermont Université, France

## Abstract

**Background:**

Puerarin, a phytoestrogen with a weak estrogenic effect, binds to estrogen receptors, thereby competing with 17β-estradiol (E2) and producing an anti-estrogenic effect. This study was to investigate whether puerarin could suppress the invasion and vascularization of E2-stimulated endometriotic tissue.

**Methodology/Principal Findings:**

The endometriotic stromal cells (ESCs) were successfully established and their invasive ability under different treatments was assessed through a Transwell Assay. Simultaneously, matrix metallopeptidase 9 (MMP-9) and tissue inhibitor of metalloproteinase 1 (TIMP-1) were detected by western blotting. Vascularization of endometriotic tissues was observed by chicken chorioallantoic membrane (CAM) assay. The staining of MMP-9, intercellular adhesion molecule 1 (ICAM-1), TIMP-1, and vascular endothelial growth factor (VEGF) in grafted endometriotic tissues was examined using immunohistochemistry analysis. The purity of ESCs in isolated cells was >95%, as determined by the fluoroimmunoassay of vimentin. E2 (10^−8^ mol/L) promoted the invasiveness of ESCs by increasing MMP-9 accumulation and decreasing TIMP-1 accumulation. Interestingly, puerarin (10^−9^ mol/L) significantly reversed these effects (*P*<0.01). The CAM assay indicated that puerarin (10^−9^ mol/L) also inhibited the angiopoiesis of endometriotic tissue stimulated by the E2 (10^−8^ mol/L) treatment (*P*<0.05). Accordingly, immunohistochemistry showed that the accumulation of MMP-9, ICAM-1, and VEGF was reduced whereas that of TIMP-1 increased in the combination treatment group compared with the E2 treatment group.

**Conclusions/Significance:**

This study demonstrated that puerarin could suppress the tissue invasion by ESCs and the vascularization of ectopic endometrial tissues stimulated by E2, suggesting that puerarin may be a potential drug for the treatment of endometriosis.

## Introduction

Endometriosis, a common, benign, estrogen-dependent disease affecting 3%–10% of women of reproductive age, is defined as the presence of endometrial glands and stroma outside of the uterine cavity [1]. It is not a malignant disorder but it has similarities to a developing tumor. For instance, endometriotic cells can be both locally and distantly metastatic, that is they can attach to, invade and damage other tissues [2], [3]. The initial phase of endometriosis is an invasive event that requires extracellular matrix (ECM) breakdown. The key enzymes that regulate the ECM are the matrix metalloproteinases (MMPs), and peritoneal invasion by endometrial tissue is thought to be dependent on MMPs and their corresponding tissue inhibitors of metalloproteinases (TIMPs). MMPs play a pivotal role in the cyclic changes of growth and tissue breakdown that occur in the endometrium. MMPs are usually synthesized during the proliferative phase and are stimulated by estrogen [4]. Several studies have shown an increase in the accumulation of MMP-1, MMP-2, MMP-3, MMP-7, and MMP-9 in endometriotic tissues. Alteration of MMP-9 and MMP-2 is an important factor in the development of endometriosis [2], [3].

Vascularization of endometriotic implants is probably one of the most important factors in the invasion of other tissues by endometrial cells. Angiogenesis is controlled by a number of inducers; of particular importance is the vascular endothelial growth factor (VEGF) family, which is significant in processes characterized by both physiological and pathological angiogenesis. Accumulation of the VEGF gene in normal human endometrial cells is acutely upregulated by E2 in vitro [5]. VEGF immunostaining has been observed in the epithelium of endometriotic implants, particularly in hemorrhagic red implants [6].

Most of the current medical treatments for endometriosis aim to downregulate estrogen activity. Historically, medical therapies have included contraceptive steroids, progestogens, and agonists of gonadotropin-releasing hormone (GnRH), as well as androgens and non-steroidal anti-inflammatory agents [7]. These treatments can only be used for a limited time because of unacceptable side effects. In addition, high recurrence rates after medical treatments are the most significant problem [8]. Therefore, exploring novel therapeutic strategies is necessary for improving the clinical management of patients with endometriosis.

Phytoestrogens exhibit estrogenic and anti-estrogenic activities both in vitro and in vivo [9]. Puerarin, a phytoestrogen derived from the Chinese medicinal herb Radix puerariae, has been proven practical in the management of various cardiovascular disorders, alcoholism, and neurological disease [10]. However, whether puerarin could suppress invasion and vascularization of endometriotic tissue is unknown.

## Results

### Establishment of the primary ectopic ESCs

**Figure 1:**
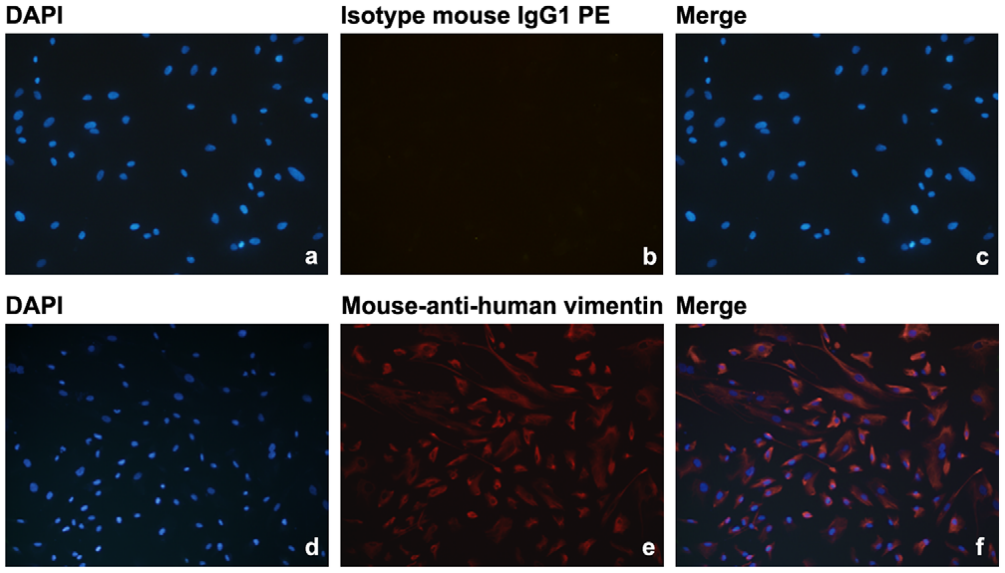
Representative photomicrographs of the fluoroimmunoassay for vimentin in the isolated ESCs. The cells were stained with DAPI (a, d), mouse IgG1 PE (b) and mouse-anti-human vimentin PE (e). The photographs (a and b; d and e) were merged on the right (c; f) respectively.

Endometriotic stromal cells (ESCs) should be stained positive with anti-vimentin-PE monoclonal antibody by fluoroimmunoassay. The negative cells could fibroblast cells, gland cells and epithelial cells. The purity of ESCs in isolated cells was >95% (Fig. 1).

### Puerarin suppresses invasion of E2-stimulated ESCs

**Figure 2:**
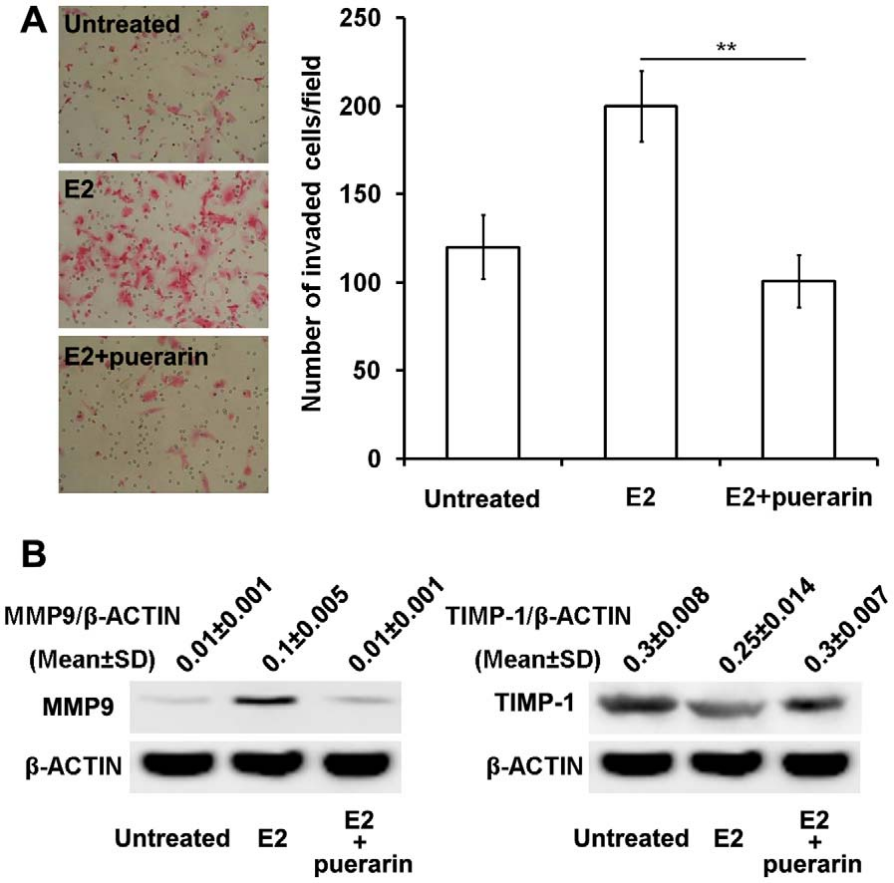
Effects of different treatments on the invasiveness of ESCs. Combination treatment of E2 with puerarin suppresses invasion (A) significantly in ESCs compared with E2 treatment alone. Western blotting (B) was performed to determine the accumulation of MMP-9 and TIMP-1 in ESCs treated with vehicle or E2 or E2 plus puerarin. **P<0.01, compared with the E2–treated cells. The results are expressed as mean ± SD, n = 6. The final concentration of E2 or puerarin was 10^−8^ mol/L or 10^−9^ mol/L respectively.

For the following experiments, 10^−8^ mol/L E2 and 10^−9^ mol/L puerarin were selected because of their efficacy on ESCs in our preliminary experiments. In Fig. 2A, 10^−8^ mol/L E2 showed a stimulatory effect on ESCs invasion compared with the untreated (vehicle) control cells, but the combination of E2 with puerarin reduced this effect (*P*<0.01).

To characterize fully the anti-estrogenic effects of puerarin, we analyzed the accumulation of MMP-9 and TIMP-1 under same treatment. As shown in Fig. 2B, the accumulation of MMP-9 was increased whereas the accumulation of TIMP-1 was decreased after the E2 treatment compared with the untreated control. With the combination of E2 and puerarin treatment, the effect was significantly reversed.

### Puerarin inhibits angiogenesis in CAM in vivo

**Figure 3:**
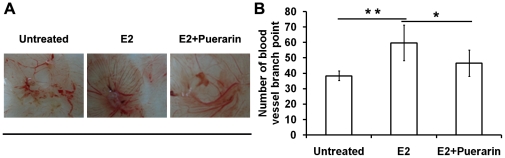
Puerarin inhibits angiogenesis in CAM in vivo. 10^−8^ mol/L E2 or combination with 10^−9^ mol/L puerarin or normal saline containing 0.1% DMSO (v/v) were loaded on gelatin sponges which were loaded on the chick chorioallantoic membranes of chick embryos respectively. After 72 h incubation, 10% v/v formaldehyde was added onto the surface of CAMs to fix the blood. The disc and surrounding CAMs were incised carefully and photographed; representative photographs show the CAMs of 10^−8^ mol/L E2 or combination with 10^−9^ mol/L puerarin or normal saline containing 0.1% DMSO (v/v) treatment groups (A). Number of visible blood vessel branch points are statistically shown (B). n = 6, * P<0.05, ** P<0.01.

To explore whether puerarin could inhibit vascularization of endometriosis tissue stimulated by E2, we tested the in vivo effect of puerarin on angiogenesis using a modified chick chorioallantoic membrane assay. E2-treated CAMs showed well-developed zones of neo-vascularization surrounding the sponge compared with vehicle treated CAM, however, CAM neovascularization was significantly suppressed by addition of puerarin (Fig. 3 A, B).

### MMP-9, TIMP-1, ICAM-1, and VEGF immunostaining of endometriotic xenograft in CAM models

**Figure 4:**
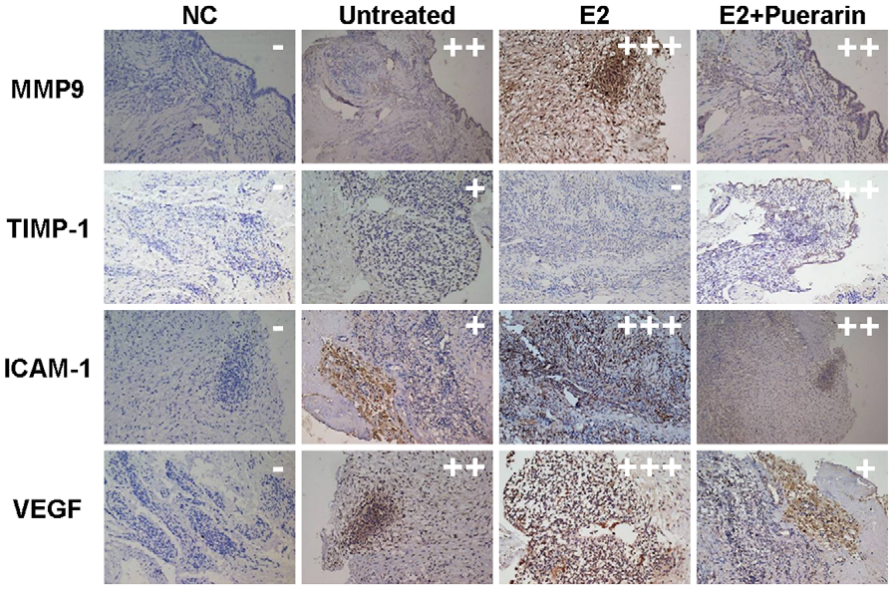
MMP-9, TIMP-1, ICAM-1, and VEGF staining of the endometriotic xenografts in the CAM model treated with vehicle, E2, and E2 + puerarin. The cells with brown color represent protein positive. E2-treated xenografts show a stronger signal for MMP-9, ICAM-1, and VEGF, and a weaker signal for TIMP-1. Co-treatment with puerarin significantly suppressed this effect. −: very weak staining; +: weak staining; ++: strong staining; +++: very strong staining. n = 6. NC: Negative control, Immunostaining for untreated group slice (omitting primary antibody).

MMP-9, TIMP-1, ICAM-1, and VEGF staining of the grafts were shown in Figure 4. Treatment with E2 resulted in a stronger MMP-9, ICAM-1, and VEGF staining and weaker TIMP-1 staining. Treatment with E2+puerarin decreased MMP-9, ICAM-1, and VEGF staining and slightly increased TIMP-1 staining compared with the E2 treatment, which suggests that puerarin could suppress E2-stimulated angiogenesis of ectopic tissues by regulating the related protein molecules.

## Discussion

Isoflavones, as phytoestrogens, are of biological interest because they exhibit estrogenic and anti-estrogenic activities both in vitro and in vivo [11]–[14]. Puerarin, the main isoflavone glycoside derived from the Chinese medicinal herb Radix puerariae, has been proven practical in the management of various cardiovascular disorders, alcoholism, and neurological disease [15], [16]. However, its role in treating endometriosis, an estrogen-dependent disorder, has not been well elucidated.

In this study, puerarin exhibits anti-estrogenic activity in a model of endometriosis. The findings support our hypothesis that puerarin could interfere with the invasion of ESCs and angiogenesis of ectopic tissues.

The establishment of endometriosis has been attributed to the attachment and invasion of retrograded endometrial fragments outside the uterine cavity. The endometriotic tissue is composed mainly of endometrial glands and stromal cells, as well as extracellular matrix (ECM). Given that the retrograde ESCs are responsible for the adherence and implantation of endometrium, ESCs rather than endometrial epithelial cells were used to assess the invasion of the retrograded endometrium [17].

MMPs can enable endometriotic tissue to digest into the ECM and underlying connective tissue. TIMPs, which affect normal and pathologic matrix remodeling, regulate the activity of MMPs. Among the different proteolytic enzymes known to degrade ECM, MMP-9 has been specifically reported to play a crucial role in the regulation of the metastatic behavior of ESCs [18]–[21]. The overexpression of MMP-9 and the low level of TIMP-1 were found in endometriotic lesions. For the most part, MMPs are synthesized during the proliferative phase and are stimulated by estrogen. In this study, we have demonstrated that ESC invasion through an artificial basement membrane was increased when treated with E2 (10^−8^ mol/L). The ability to invade the basement membrane and to migrate in response to the E2 stimulus was reduced after co-treatment of ESCs with puerarin (10^−9^ mol/L). Moreover, western blot showed that the alteration of MMP-9 and TIMP-1 level was the possible mechanism for the anti-metastatic action of puerarin.

Endometriosis requires estrogen for its continued growth and, if deprived of this hormone, it tends to regress. Estrogen plays a key role in stimulating adhesion, invasion, and implantation of menstrual endometrial fragments through its action on estrogen receptors (ER). Reportedly, puerarin can treat endometriosis partly by suppressing P450arom, a key enzyme for estrogen biosynthesis [22], [23]. Another study showed that phytoestrogens act as aromatase inhibitors at low-concentrations [24].

We also focused on the suppression of endometriotic tissue angiopoiesis by puerarin. Preparation of stromal cells and epithelial glands cannot form endometriotic-like lesions in the CAM model [25]. This occurs only when the tissue architecture of the endometrium is preserved [26]. In the CAM model, the human endometrium was observed as highly angiogenic and, therefore, perfectly capable of stimulating blood vessel formation in the surrounding tissues. The phenomenon was more pronounced when treated with E2. This positive effect can be suppressed by co-treatment with puerarin. Related proteins, including ICAM-1, TIMP-1, MMP-9, and VEGF, are involved in the process.

Angiogenesis is a vital process in the development of endometriotic implants, providing substantial background for their further maintenance and growth. High levels of estrogen in the microenvironment of endometriotic lesions stimulate specific angiogenic growth factors such as VEGFs and ICAM-1. ICAM-1 has previously been linked to VEGF-dependent revascularization, and they both play important roles in pathological process in endometrial tissue [27], [28]. Our data show 10^−9^ mol/L of puerarin can significantly inhibit the effects of estrogen on angiogenesis during lesion implantation.

The current standard medical treatments for endometriosis include GnRH agonists, contraceptive steroids, progestogens, and androgens [29]–[33], all of which aim to lower circulating E2 concentrations. Of these agents, GnRH agonists appear are the most effective, but they are expensive and long-term treatment is not possible because of the loss of bone mineral density. Progestogens have the best clinical profile and a good cost-effectiveness balance; however, most studies found that they are not as effective as GnRH agonists. Oral contraceptives are only effective during treatment and have a high relapse rate after therapy is completed. Markedly high recurrence rates of up to 45% after current medical or surgical therapy have been reported [34]. Therefore, exploring new strategies for treating endometriosis is needed.

Clinically, the potential advantages for puerarin to treat endometriosis are as follows; first, puerarin is able to bind to ER-alpha and ER-beta [9], it can suppress P450arom activity as aromatase inhibitors in endometriosis treatment, and it may be applicable as an adjuvant to the current medical treatments. Second, because puerarin can be used for long periods without severe side effects, medical treatment with this agent is a good option for avoiding disease relapse after the initial surgical and/or medical therapy. Third, with weak estrogen agonists/antagonists and some other enzymatic activities, puerarin is increasingly advocated as a natural alternative to estrogen replacement therapy and is available as dietary supplements.

In summary, we demonstrated that puerarin has anti-estrogenic activity by interfering with the invasion of ESCs and angiogenesis of ectopic tissues. These findings suggest that puerarin may be a new and efficient product for the medical treatment of endometriosis.

## Materials and Methods

### Tissue collection and cell culture

ESCs were obtained from premenopausal patients who had undergone salpingo-oophorectomy or evisceration for ovarian endometriotic cysts (n = 6). All patients had been free of any hormonal treatments before the operation. All the samples were obtained in the proliferative phase of the cycle, which was confirmed histologically. This study was approved by the human investigation committee of the Changhai Hospital, and written informed consent was obtained from all patients.

The tissues were collected under sterile conditions and transported to the laboratory on ice in Dulbecco's modified Eagle's medium (DMEM) (PAA, Linz, Austria). The ESCs were isolated as described by Kaei Nasul [8] and cultured in flasks with DMEM containing 10% charcoal stripped fetal bovine serum (FBS) (Biological Industries, Israel), 100 IU/mL penicillin, and 100 IU/mL streptomycin, which were then incubated in 5% CO_2_ at 37°C. For CAM experiments, tissue specimens were minced to fragments of 2×2 mm using a scalpel in a sterile petri dish. Tissue fragments that appeared to be necrotic were discarded.

The purity of ESCs in isolated cells cultured on a cover slip was determined by fluoroimmunoassay of vimentin (mouse anti-vimentin-PE monoclonal antibody, Thermo, Germany) as previously described [35]. Isotype mouse IgG1 PE (eBiosicence, USA) was used as the control. The purity was calculated by vimentin-stained cells in comparison with DAPI-stained cells.

### Invasion assays

ESCs invasion was assayed in transwell chambers (Costar, USA) according to the manufacturer's instruction. Briefly, transwell chambers with 6.5 mm polycarbonate filters of 8-µm pore size were used. ESCs were starved overnight in serum-free medium containing 0.1% BSA prior to initiation of the invasion assay. The upper surface of the filter was coated with 20 µl 10 mg/ml Matrigel (Beckon Dickinson Labware, USA). The number of cells was adjusted to 5×10^5^/ml in DMEM containing 0.1% charcoal stripped BSA, and 5×10^4^ cells was added to the top well after Matrigel was polymerized. 600 µl DMEM containing 10% charcoal stripped FBS was added to the bottom well. Cells were treated with 1×10^−8^ mol/L E2 (Sigma, NY, USA) or a combination with 1×10^−9^ mol/L puerarin (Sigma, NY, USA) or normal saline containing 0.1% DMSO (v/v) for 12 h, the non-invaded cells were completely wiped out with a cotton swab, and the lower surface of the filter was fixed in methanol, stained with HE, and counted under a microscope at a magnification of 200× with a Leica Qwin system (Leica, Germany). For each test, the cells in 5 randomly selected fields were determined, and the counts were averaged. The results were from 6 independent experiments (n = 6).

### Western blot analysis

To clarify whether puerarin could affect the levels of MMP-9 and TIMP-1 protein, ESCs were treated with above treatments at 37°C for 48 h. After the treatment procedures, cells were washed by pre-cooled PBS and lysed RIPA buffer (50 mmol/L Tris–Cl pH 7.4, 150 mmol/L NaCl, 1% NP-40, 0.1% SDS, 0.5% sodium deoxycholate) supplemented with 1 mmol/L PMSF. Protein concentration was determined using the BCA™ protein assay kit (Pierce, IL). 30 µg protein samples were resolved by SDS-PAGE and transferred to a polyvinylidene difluoride membrane (Millipore, MA). MMP-9 and TIMP-1 protein were detected by immunoblot with anti-MMP-9 and anti- TIMP-1 polyclonal antibody (Santa Cruz, CA) at a 1∶500 dilution or β-actin (1∶5000; Sigma, USA), then followed by incubation with peroxidase-coupled secondary antibodies. A Supersignal kit (Pierce, IL) was used to visualize the bands according to the manufacturer's instructions. Six independent experiments were assessed (n = 6).

### Chick Chorioallantoic Membrane (CAM) Assay

The in vivo puerarin anti-estrogenic effect on angiogenesis of ectopic lesions was evaluated using the CAM vessel development assay with modifications as previously described [36]. Fertilized chicken eggs were incubated in a standard egg incubator at 37°C and 60%–70% relative humidity for 6 days. A circular window 1.5–2 cm in diameter was opened aseptically on the eggshell, and minced tissue fragment was grafted into CAM. The window was sealed with Para film and the eggs were replaced to the incubator. After 24 h, the eggs were randomly assigned into three groups and treated with a gelatin sponge (4×4×4 mm) which was saturated with 10^−8^ mol/L E2 or combination with 10^−9^ mol/L puerarin or normal saline containing 0.1% DMSO (v/v). After 72 h the area around the loaded gelatin sponge was photographed using a Canon digital camera. The angiogenic index was defined as the mean number of visible blood vessel branch points within the defined area of the gelatin sponge. Assays for each test sample were carried out using 6 eggs.

### Immunohistochemistry assay

For immunohistochemistry, 5 µm paraffin sections of CAM xenograft tissues were prepared and pretreated at 65°C for 2 hours, followed by deparaffinization. Antigen retrieval was carried out before application of the primary antibodies (MMP-9, TIMP-1, ICAM-1, and VEGF; 1∶1000 dilution; Santa Cruz, CA, USA) overnight at 4°C. Thereafter, the slides were incubated for 2 hours at room temperature with the secondary antibodies conjugated with horseradish peroxidase (HRP; 1∶100; DAKO, Japan). HRP activity was detected using the Liquid DAB+Substrate Chromogen System (DAKO, Japan). Finally, the sections were counterstained with hematoxylin and photographed.

### Data and Statistical Analysis

Data were represented as mean ± SD. Statistical comparisons between groups performed using 1-way ANOVA followed by Student's t-test. *P*<0.05 was considered statistically significant.
